# Influence of Publicity and Education and Environmental Values on the Green Consumption Behavior of Urban Residents in Tibet

**DOI:** 10.3390/ijerph182010808

**Published:** 2021-10-14

**Authors:** Huifang Ma, Weidong Chen, Hailin Ma, Hude Yang

**Affiliations:** 1 College of Management and Economics, Tianjin University, Tianjin 300072, China; hfm790105@126.com; 2 Politics and Public Administration College, Qinghai Nationalities University, Xining 810007, China; qhmyyhd@163.com; 3 Plateau Brain Science Research Center, Tibet University, Lhasa 850000, China; hailin_ma79@163.com

**Keywords:** publicity and education, environmental values, price sensitivity, green consumption behavior, Tibet

## Abstract

Faced with ecological environmental issues and a surge in the consumption of products in the Qinghai–Tibetan Plateau, it is necessary to explore the effective driving mechanism of green consumption behavior. This study investigated the impact of publicity and education on green consumption behavior and explored the mediating effects of environmental values and the moderating effects of price sensitivity. A cross-sectional survey was conducted with a sample of 500 questionnaires, which were randomly distributed by stratified random sampling to municipal government departments, public institutions, communities, streets, shops, and supermarket entrances in Lhasa, Shan Nan, and Xigaze in Tibet. Further, structural equation modeling was applied to derive data for statistical analyses. Publicity, education, and environmental values had a significant influence on green consumption behavior. Environmental values play a mediating role in the influence of publicity and education on green consumption behavior. Price sensitivity negatively moderates the relationship between publicity and education and environmental values; when considering the price of green consumption, the positive impact on environmental values is weakened by publicity and education. Furthermore, it moderates the mediating effect of environmental values. Publicity and education remain the primary intervention for promoting green consumption. Especially in areas with ethnic minorities, publicity and education in combination with the characteristics of ethnic areas should be used to promote the traditional Tibetan culture of respect for all life and to live in harmony with nature. Moreover, policies, regulations, and tax subsidies related to green consumption should be improved while reducing the negative impact of prices and other economic factors on the propensity to consume. These findings provide empirical evidence for the complex relationship between government intervention measures and urban residents’ green consumption behavior.

## 1. Introduction

The Qinghai–Tibetan Plateau is ecologically vulnerable to human activities and environmental changes, and it is very difficult to restore the region once destroyed [1]. It has a unique ecology and geography along with diverse ecosystem types. Many important rivers originate in the Qinghai–Tibet Plateau, such as the Yangtze, Yellow, and Mekong rivers [2,3]. As Asia’s water tower, the Qinghai–Tibetan Plateau plays a key role as a barrier to the ecological security of China and East Asia. Therefore, ecological changes in the Qinghai–Tibet Plateau can have large consequences on the local and surrounding regions.

Tibet is a primary part of the Qinghai–Tibet Plateau. In recent years, the developments in the social economy; improvements in traffic conditions, such as roads and railways; and the continuous growth of populations coupled with surges in the consumption and tourism industry have contributed to ecological environmental problems in Tibet, which have been extensively studied by researchers [4]. The unsustainable crisis in the process of economic development and environmental pollution is often accompanied by excessive carbon emissions and anthropogenic activities, resulting in substantial eco-environmental problems [5]. The magnitude of global warming in recent decades on the Tibetan Plateau, the largest plateau in the world, has surpassed the average of the Northern Hemisphere [6]. Since 2018, 8.4 tons of garbage have been collected from elevations above 5200 m in the Mount Everest Nature Reserve, which has attracted widespread public concern. Schipper (1989) pointed out that in industrialized countries, about 45–55% of the total energy use is influenced by consumers’ activities for personal transportation, tourism, personal services, and homes [7]. Green consumption requires consumers to avoid or reduce damage to the environment, reduce carbon emissions, pay environmental premiums for green products, and change their original consumption habits, which is undoubtedly the simplest, scalable, and fundamental strategy for ecological environmental protection.

Green consumption plays a crucial role in reducing the negative impact of consumption on the environment and ecosystems [8]. It is based on consumer health protection and resource conservation and conforms to the long-term benefits of society while focusing on sustainable consumption and green environmental protection behavior. Recently, research on green consumption has increased remarkably, attracting attention from scholars in interdisciplinary studies [9]. Most of the existing research also focuses on the influencing factors and driving mechanisms of green consumption behavior. Guo (2020) examined the impact of social exclusion (i.e., being rejected, isolated, excluded, or ignored by other individuals or groups in society) on consumers’ intention to consume green [10]. Ge (2020) explains the internal influence mechanism between social norm conflict and green consumption [11]. Amatulli (2019) indicates that under the influence of expected shame, negatively framed messages are more effective than positively framed ones in prompting consumers to engage in pro-environmental behaviors [12]. Researchers define green consumption behavior in the following way: “After becoming aware of environ mental problems, individuals consider their own interests and reduce the loss to the environment through consumer behavior, including buying, use management, and processing of waste, linking all three” [13,14]. Promoting green consumption not only stimulates sustainable industrial development but also improves resource utilization efficiency. Li [15] reviewed previous studies on green consumption and classified the influencing factors into five categories: consumers’ own factors, objective factors, the psychology and lifestyle of green consumption behavior, self-interest, and social interest. Recently, “high green consumption preference” and “low green consumption behavior” are interesting areas of research for green connection [16]. Existing studies have designed to close the gap mainly by changing individual cognition by highlighting environmental protection [17], personal health, and social norms to promote green attitude changes to green behavior [18,19]. One study suggested that strong attitudes are more likely to affect behavior, while weak attitudes are more likely to be shaped by behavior [20]. If we want to see a greater commitment to green consumption practices, government bodies need to play a more active role [21]. Publicity and education are considered to be the most effective interventions for cultivating green consumption attitudes and changing public consumption habits [22]. However, these interventions are also influenced by economic and other objective factors [23].

Understanding the ecological consequences of climate change and anthropogenic activities in Tibet, the Chinese government strives for achieving the ecological security of Tibet [24]. In recent years, the central and local governments have executed a large amount of environmental protection works and hae advocated for green and low-carbon life in the whole region, but these measures have had an insufficient effect [25,26]. Hence, further exploration of the mechanism of green consumption behavior at the microlevel is necessary. In this study, we focused on how publicity and education change the internal cognition of urban residents of Tibet to promote their green consumption behavior. Since green products are more expensive than ordinary products, they require consumers to pay an environmental premium. Will concerns about price affect their acceptance of green consumption publicity and education, and thus weaken their green consumption behavior?

The theory of planned behavior (TPB) is one of the most influential behavioral theories and has been used in studies on green consumption behavior [27]. According to the theory, individual behaviors are thoughtful and planned, and various factors indirectly affect individual behavioral decisions through willingness. Based on the above questions and guided by TPB, we built a theoretical model; in this model, publicity and education was the independent variable and green consumption behavior was the result variable, while environmental values and economic factors were also considered. A stratified random sampling survey was conducted in the Tibet Autonomous Region to collect data and verify the model.

## 2. Review of Literature and Hypothesis Development

### 2.1. Influence of Publicity and Education

Publicity and education enable people to have a certain understanding, recognition, analysis, and choice of what to do in advance [8]. Publicity and education related to the ecological environment can help individuals to establish appropriate environmental values. Encouraging citizens to adopt more environmentally friendly consumption habits has become a critical challenge faced by government agencies and policymakers. The local government of the Tibet Autonomous Region has firmly implemented the green development strategy and the most stringent environmental protection policies [28]. In 2013, “the measures of the Tibet Autonomous Region for the supervision and management of ecological and environmental protection” were promulgated and implemented. It rules for ecological and environmental protection in Tibet in terms of protection and development, rewards, and punishments. During the annual “National Energy Conservation Publicity Week” and “National Low Carbon Day,” the local government holds a series of activities, such as “Beautiful Tibet Low Carbon Action” and “Low Carbon Travel” to create awareness and educate the populace about the concept of environmental protection and to advocate for the implementation of green and low-carbon life in the region. A study of household energy behavior in Sweden found that education significantly improved household energy use behavior [29]. A research on the garbage classification of urban residents found that the transmission of classification information by the government and the activities of publicity and education can effectively enhance residents’ awareness of garbage classification and can influence their final behavior choice [30]. Therefore, publicity and education for green consumption will not only improve public awareness and evaluation of environmental issues directly but can also form a green consumption social atmosphere, thus guiding individual behavior for environmental protection. Based on the above analysis, this study proposed the following hypotheses:

**Hypothesis** **1** **(H1).** *publicity and education positively affect environmental values.*

**Hypothesis** **2** **(H2).** *publicity and education positively affect individual green consumption behavior.*

### 2.2. Environmental Values as a Mediator between Publicity and Education and Green Consumption Behavior

An individual’s cognition and experience attitude about the environment will inevitably affect the way they interact with the natural environment. Previous studies confirm that there is a close relationship between environmental values and environmental behaviors [31,32]. Influenced by natural conditions and religious values, people living on the Qinghai–Tibet Plateau have naturally developed a simple ecological ethics where they value nature, revere nature, and live in harmony with nature [33,34]. Cultural norms shape behavior [35]. For a long time, in a special living environment, Tibetans have formed a unique traditional culture and an ecological and ethical concept that has a large influence on the society. Individuals’ value judgment and sense of responsibility for environmental issues are important factors that lead to environmentally friendly behaviors. When individuals believe that they are responsible for environmental pollution and ecological problems, they are more likely to engage in environmentally friendly behaviors [36]. Stern [37] and other scholars have confirmed that consumers’ environmental values have a key effect on their environmental attitudes and behaviors. This study considers that under the influence of Tibetan traditional culture, urban residents in Tibet have more positive environmental values, which can make them accepting of green publicity and education more easily and can influence their behavior; thus, environmental values can positively predict individuals’ green consumption behavior and can have a mediating effect in the process of publicity and education by positively affecting individuals’ green consumption behavior. Based on this, the following hypotheses were proposed:

**Hypothesis** **3** **(H3).** *environmental values positively influence green consumption behavior.*

**Hypothesis** **4** **(H4).** *environmental values have a mediating effect on the influence of publicity and education on green consumption behavior.*

### 2.3. Adjusting Effect of Price Sensitivity

Price behavior is of primary importance in economics because it is the target of the central bank’s monetary policy [38]. Rational individuals strive to maximize their utility (i.e., including shopping, nutritional, and other material needs, pleasure, convenience, etc.) within the limits given by a budgetary constraint. Therefore, they often exhibit some degree of reactivity or sensitivity to price. Neoclassical theory provides a rich set of testable implications for how consumer demand responds to changes in relative prices and income [39]. Usually, price responsiveness is measured by “price elasticity” and “price sensitivity.” Price elasticity measures consumers’ sensitivity to changes in real prices, holding the real income constant. Price sensitivity focuses on an individual’s response to price and price changes and is measured from operational dimensions such as “price importance,” “price consciousness,” and “price willingness to pay” [40,41].

Price sensitivity extends to the field of green consumption, which refers to the sensitivity of individuals to economic factors, such as green products and services. The mechanism by which price sensitivity influences green consumption behavior needs to be further explored [41]. Green products and services are expensive because they involve more technology and cost factors than ordinary products. Moreover, the impact of economic factors on individuals who receive green environmental education and establish environmental value experiences is uncertain. Cicia et al. found that up to 78% of respondents indicated that they preferred organic products in real life but would only buy them if their price did not exceed 20% of the price of general agricultural products [42]. Other studies also indicated that financial constraints and the concern for costs and benefits are one of the key reasons for people not participating in socially responsible consumption [43]. Studies have also noted that tax and subsidy policies also have a significant positive effect on residents’ energy conservation behavior; the higher price level of green products is related to the phenomenon of an attitude and behavior gap in green consumption [23,44]. Notably, the considerations of price influence the attitude and experience of the individual. Although publicity and education aid in cultivating the right environment values, prices and other economic factors influence the individual’s response to environment problems, thus affecting their environmental behavior; this pattern is also observed in Tibet. Based on the above analysis, the following hypotheses was proposed:

**Hypothesis** **5** **(H5).** *price sensitivity has a negative moderating effect on the relationship between publicity and education and environmental values; when more consideration is given to the price of green consumption, the positive impact of publicity and education on environmental values is weakened, and the mediating effect of publicity and education on green consumption behavior through environmental values is weakened, and vice versa.*

## 3. Sampling and Methodology

### 3.1. Measurement Scales

All measurement scales used in this study were based on the existing mature scale and were adjusted according to the specific cultural background. The publicity and education scale was adapted from Lee [45]. Four questions were used to test residents’ perceptions of publicity and education about green consumption knowledge and its influence on themselves. The environmental values scale was adapted from Dunlap et al. [46], which included the seriousness of environmental problems and the cognition of the relationship between environment and individual behavior, and finally formed seven items after adjustment. The price sensitivity scale adapted from Van Doorn and Verhoef [47] was used to investigate the influence of economic factors on individual green consumption behavior. The green consumption behavior scale was adapted from Lee et al. [48], including the three dimensions of purchase, use management, and recycling, with a total of 10 items and individuals’ green behaviors in daily eating, clothing, use, transportation, and purchases were investigated. A 5-level Likert scale was used for all questionnaires, with 1–5 indicating completely inconsistent, relatively inconsistent, uncertain, relatively consistent, and very consistent categories, respectively. The five major components of the questionnaire were personal information, scales on publicity and education, environmental values, price sensitivity, and green consumption behavior. These four instruments are outlined in Appendix A.

### 3.2. Sample Selection and Data Collection

This study adopted a cross-sectional design. The reliability and validity of the questionnaire were tested through a small-scale pre-investigation, and a formal questionnaire was obtained after revisions. A formal survey was conducted in July 2019. A total of 500 questionnaires were randomly distributed by stratified random sampling to municipal government departments, public institutions, communities, streets, shops, and supermarket entrances in Lhasa, Shan Nan, and Xigaze of Tibet. A total of 493 questionnaires were collected; 42 invalid questionnaires were excluded; and 451 valid questionnaires were retained, with an effective rate of 91%. Among them, 188 were male (43.1%) and 248 were female (56.9%) participants. Several demographic variables contained missing values. Table 1 lists all variables used in the study.

## 4. Results

**Figure 1:**
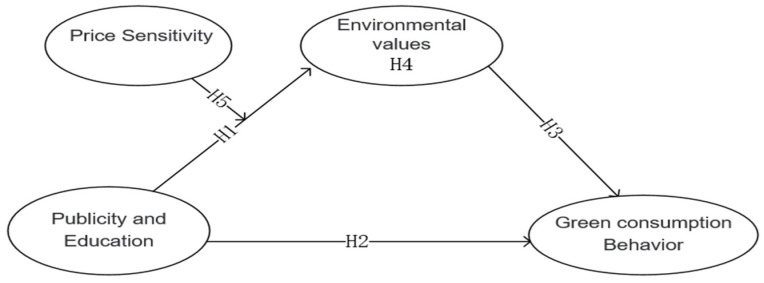
Conceptual model of the study. Note: H1 publicity and education positively affect environmental values.H2 publicity and education positively affect individual green consumption behavior.H3 environmental values positively influence green consumption behavior. H4 environmental values have a mediating effect on the influence of publicity and education on green consumption behavior.H5 price sensitivity has a negative moderating effect on the relationship between publicity and education and environmental values; when more consideration is given to the price of green consumption, the positive impact of publicity and education on environmental values is weakened, and the mediating effect of publicity and education on green consumption behavior through environmental values is weakened, and vice versa.

The proposed theoretical model (Figure 1) was tested using structural equation modeling to gain a more robust understanding of the interdependent relationships between the various constructs. Structural equation modeling is a widely used multivariable statistical method in the field of behavior research and social science because of its high statistical efficiency and flexibility. In structural equation models, it is conventional to divide the modeling into two parts: a measurement model and a structural model [49]. Initially, the measurement model containing all latent constructs and indicators was analyzed, and then the hypothesized structural model was tested.

### 4.1. Reliability and Validity Test of Scale

Statistical analysis software (AMOS 23.0) (IBM, Armonk, NY, USA) was used to verify the reliability, convergent validity, and discriminant validity of the questionnaire scale with confirmatory factor analysis. First, the Cronbach’s α coefficient of each variable ranged from 0.777–0.906, which indicated that the variables used had good reliability. Second, confirmatory factor analysis was conducted for each variable, and the analysis results showed that the load of each factor was greater than 0.700; the compound reliability of all variables was >0.7, and the average variation extracted (AVE) was >0.5. This indicated that the variables in this study had good reliability and validity (see Table 2).

The correlation coefficient, and square root of the AVE of each variable are shown in Table 3.

A significant correlation was observed between the variables (*p* < 0.01). Moreover, to test the possible homologous variance problem, in Harman’s single factor method, the SPSS software (version 23.0) (SPSS, Inc., Chicago, IL, USA) was used to conduct exploratory factor analysis. The variance interpretation amount of the initial eigenvalue of the first factor without rotation was 31.35% and did not exceed 50%. Comparing the AVE square root of each variable and the Pearson correlation coefficient between the variables revealed that the AVE square root was greater than the Pearson correlation coefficient between itself and other variables; this indicated that all variables had discriminative validity and that there was no serious common method deviation.

### 4.2. Overall Structural Model and Research Hypothesis Testing

The overall model fitting was verified, and the model fitting indexes were obtained. The chi-square value of fitness was 196.674, df = 73, X^2^/df = 1.906, indicating that the theoretical model can be adapted to the observed data. Additionally, the overall fitness of the model with results (RMSEA = 0.045, RMR = 0.026, IFI = 0.998, CFI = 0.998, NFI = 0.997, GFI = 0.998) showed that the hypothesis model can fit with the observed data.

#### 4.2.1. Main Effect Test

The AMOS23.0 software was used to test the direct causal relationship between the three variables: publicity and education (PE), environmental values (EV), and green consumption behavior (GCB) and to verify the hypothesis. The results are presented in Table 4. Publicity and education had a significant positive influence on environmental values (β = 0.361, *p* < 0.001); thus, these results supported Hypothesis 1. Publicity and education had a significant positive effect on green consumption behavior (β = 0.179, *p* < 0.01); this was consistent with Hypothesis 2. Environmental values had a significant positive effect on green consumption behavior β = 0.159, *p* < 0.01), which supported Hypothesis 3. Environmental values positively influenced green consumption behavior.

#### 4.2.2. Mediating Effect Test

The bootstrap confidence interval method was used to test the mediating effect.

The bootstrap sampling was set to 2000 times. If the percentile confidence interval of the indirect effect did not contain 0 at the 95% confidence level, it indicated that there was a mediating effect; otherwise, there was no mediating effect. The results of the mediating effect test in Table 5 show that the 95% confidence interval (CI) of the total effect for PE and GCB was 0.253 and 0.494; the indirect effect 95% CI was 0.040 and 0.231, whereas those of the direct effect 95% CI were 0.099 and 0.397; both the results did not contain zero, which indicated the existence of a partial mediating effect. This demonstrated that Hypothesis 4 was valid, and environmental values have a mediating effect on the influence of publicity and education on green consumption behavior.

#### 4.2.3. Moderated Mediating Effect Test

The bootstrap confidence interval method was used to test the moderating effect of the model. All variables were standardized, and all operations were computed using PROCESS v3.2 (macro program in SPSS). Model 7 was selected in SPSS23.0, and random sampling was set to 2000 times at a 95 % confidence interval. When the confidence interval of the interaction term between the independent variable and the regulating variable did not contain zero, it indicated that a regulating effect exists; otherwise, there was no regulating effect. The results show that the interaction between publicity and education and price sensitivity had a significant negative moderating effect on environmental values (β = −0.202, *p* = 0.000) (Table 6). At different levels of moderating variables, the 95% confidence interval did not contain zero (Table 7). Specifically, price sensitivity negatively moderated the first half of the mediating effect.

To test the essence of the interaction effect, the price sensitivity average, plus or minus one standard deviation, was categorized into a high price sensitivity group (more economic factors considered) and a low-price sensitivity group (fewer economic factors considered). Notably, under different values of green consumption, publicity and education had different influences on environmental values.

The moderated mediating effect test results showed that when the value of price sensitivity was −1SD, the 95% confidence intervals of the bootstrap test were 0.040 and 0.189 for the mediating effect of publicity and education on green consumption behavior through environmental values, respectively. When the value of price sensitivity was +1SD, the 95% confidence intervals of the bootstrap test were 0.017 and 0.079 for the mediating effect of publicity and education on green consumption behavior through environmental values, respectively (Table 8), which indicated that the moderated mediating effect was established. Specifically, at different values of price sensitivity, publicity and education had different influences on green consumption behavior through environmental values. With the increasing influence of price sensitivity, the effect of publicity and education on green consumption behavior through environmental values was weakened, thus, verifying Hypothesis 5.

## 5. Discussion

The protection of the ecological environment has become a key strategic goal of the Chinese government. Green consumption is one of the primary ways in which human actions can protect the environment. This study explored the factors influencing green consumption behavior and the possible psychological mechanisms of the moderating effect. The variables involved in the study included publicity and education, environmental values, price sensitivity, and green consumer behavior. The results confirmed that there is a direct or indirect relationship between these variables. Intriguingly, the existence of relationships between publicity and education and environmental values or green behavior may be weakened in the presence of other explanatory factors such as price sensitivity. The findings of this study can act as a reference for both environmental regulators and public policymakers who want to promote green consumption behavior and an increasing ecological civilization construction.

As hypothesized, publicity and education are a strong antecedent of green consumer behavior that should not be excluded from studies. According to the theory of communication and persuasion, publicity and dissemination of relevant information on a certain aspect will affect an individual’s internal cognition and behavior. The factor in the communication or persuasion effect depends on “attitudes toward the communicator” and “the cues which elicit them” [50]. The qualitative study performed by Han et al. suggested that in addition to the negative green perceptions, a lack of justification and a lack of social awareness are characteristics of individuals exhibiting green gap behavior [51]. To overcome this gap, consumers should be placed in contact with green consumers. Our research also confirmed that publicity and education have a significant positive effect on green consumption behavior. Publicity and education will allow individuals to understand green consumption and improve their desire to participate actively, which will change the cognitive construction of individuals to a certain extent and further affect their related behavior.

Yearly, the influx of tourists to Tibet contributes to the development of economy and society in Tibet. Although the development of tourism increases the income of urban residents, it also creates a series of problems to the local environment. Higher productivity translates into higher income, resulting in increased consumption, which further leads to excessive production of waste from increased consumption [52]. Environmental publicity and education in Tibet should focus on these changes and avoid issues, such as extravagant consumption, conspicuous consumption, and an unbalanced consumption structure. This may be possible by implementing targeted publicity and education activities, especially targeting people who come to Tibet for tourism or business, or urban residents whose spending power is increasing, and encourage them to practice green lifestyles while travelling and shopping.

According to the theory of environmental consciousness, environmental values and awareness are important influencing factors of green consumption [22]. In enterprises, when employees realize the seriousness and importance of environmental problems, they will take corresponding environmental protection actions, and the intuitive benefit is to reduce the waste of resources and save operating costs [53]. Cultural, social, and religious values are key to individual decision-making in this region. Based on the ethical values hypothesis proposed by Kaplan, religions play a major role in shaping human ethical and cultural value systems across the world, and Mostafa confirmed that religious participation fosters altruistic and pro-environmental behaviors [54]. Cultural factors moderate many aspects of consumers’ impulsive buying behavior, including self-identity, normative influences, suppression of emotion, and postponement of instant gratification [55]. Environmental propaganda work should use national and regional characteristics, explore the connotation of environmental protection from the traditional Tibetan culture, and impart the characteristics of the public through cultural propaganda. This approach will help to improve individual environmental value and enhance individual green consumption behavior. Social psychology proves that persuasive information must first be understood and internalized before it can play a role in individual behaviors. Nevertheless, environmental pollution caused by some religious ceremonies and folk activities, such as mulberry feeding, should be deeply considered [56]. With utmost respect to religious practice, the concern is about pollution control and devotees’ health. The public should adopt more scientific and reasonable ways to express the symbolic meaning of religion [57].

Additionally, the study found that price sensitivity negatively moderates the path of publicity and education and environmental values on green consumption behavior. We found that this was affected in the first half of the model, and it further moderated the mediating effect of environmental values on green consumption behavior. Economic theory holds that individual behavior can be understood in terms of the goal of utility maximization; therefore, based on price and income, consumption choices can be predicted [58]. Lee Weisstein et al. [59] reported that price framing (gain vs. reduced loss) moderates the relationship between consumers’ green perceptions and their intention to buy green products. Litvine and Wüstenhagen analyzed the behavioral factors directly influencing green purchases and highlighted that the role of willingness to pay is a major factor directly affecting green behavior [60]. Moreover, perceived simplicity and benefit certainty indirectly affect behavior by mediating intentions [61]. Barbarossa and Pastore considered that in a marketplace setting, green consumers tend to be more conscious of the price, quality, and lack of availability of green products [62]. Presently, with the rapid development of the economy, the consumption and purchasing of power by urban residents in Tibet have increased significantly. However, a significant observation was that the per capita income of the majority of middle-class and low-income population is relatively low. Hence, a considerable number of consumers are more willing to choose non-green products at a lower price than opt for green organic products with high prices. Thus, the dominance of self-interest values over environmental values leads to an increase in high environmental attitudes and low environmental behavior. Therefore, it is necessary to fully consider the restrictions and influence of economic factors on green consumption behavior. Policies and regulations should be constantly improved and the compensation for green products should be increased.

## 6. Conclusions

Green consumption is an emerging environmental topic that is receiving increasing attention. In the current study, we constructed a structural equation model based on the theory of planned behavior to explore the driving mechanism of green consumption behavior. Our study also verified that publicity and education is the most effective measure to promote green consumption behavior in ethnic minority areas. However, environmental values play a partial mediating role in the influence of publicity and education on green consumption behavior. This is consistent with previous assumptions and predictions. In Xizang, many local people believe in Tibetan Buddhism and are deeply influenced by Tibetan culture; their belief makes them feel accountable for environmental destruction and pollution. Therefore, publicity and education in combination with the characteristics of ethnic areas can be used to promote the traditional Tibetan culture of respect for all life and to live in harmony with nature. This allows enhancement of an individual’s internalization of the values and stipulations of the environments, and thus, it can significantly promote green behavior. Finally, we should fully consider the restriction and influence of economic factors on green consumption behavior, constantly improve policies and regulations, and increase the compensation for green behavior.

In this study, environmental values and price sensitivity were selected as the mediating and moderating variables of the model. The results of data analysis confirmed our hypotheses. However, individual behavior is the result of complex internal and external interactions. There may be other potential mediating variables in reality, especially in ethnic areas. Cultural factors such as religion, beliefs, and customs are worth considering. Therefore, there is still room for further model enrichment and extension.

## Figures and Tables

**Table 1:** Sample’s sociodemographic profile (%), *N* = 451.

**Variable**	**Category**	**Number of People**	**Percentage (%)**	**Variable**	**Category**	**Number of People**	**Percentage (%)**
Marital status	Married	310	68.9	Education	High school degree or below	49	10.9
	Not married (widowed/divorced/separated)	129	28.7		Junior college and undergraduate	316	70.4
	Other	11	2.4		Postgraduate and above	84	18.7
Age	<30	177	40.0	Residence	Urban	331	73.9
	31–40	164	37.1		Suburban	77	17.2
	>40	101	22.9		Rural	40	8.9
Annual household income	<50,000 RMB	51	12.0	Employment	Civil servant	94	21.1
	50,000-100,000 RMB	110	25.9		teacher	213	47.8
	100,000–150,000 RMB	71	16.7		Individual business	29	6.5
	150,000–200,000 RMB	94	22.2		Worker	28	6.3
	>200,000 RMB	98	23.1		Other	82	18.4

**Table 2:** Confirmatory factor analysis and reliability analysis results of variables.

Latent Variable	Indicator	Standardized Factor Loading	CR	AVE	Cronbach’s α
PE	XB1	0.700	0.817	0.528	0.815
	XB2	0.759			
	XB3	0.732			
	XB4	0.714			
PS	XH1	0.741	0.778	0.539	0.777
	XH2	0.709			
	XH3	0.751			
GCB	GMGZ	0.747	0.813	0.593	0.812
	SYGL	0.797			
	HSLY	0.765			
EV	XD1	0.798	0.909	0.588	0.906
	XD2	0.786			
	XD3	0.758			
	XD4	0.792			
	XD5	0.723			
	XD6	0.750			

Note: PE: publicity and education; PS: price sensitivity; GCB: green consumption behavior; GMGZ: purchase; SYGL: use management; HSLY: recycling; EV: environmental values; CR: compound reliability; AVE: average variation extracted.

**Table 3:** Discriminant validity of potential variables.

	PE	EV	PS	GCB
PE	0.739			
EV	0.515 **	0.767		
PS	0.261 **	0.265 **	0.734	
GCB	0.347 **	0.319 **	0.450 **	0.770

Note: ** represents a significant correlation at the level of 0.01 (bilateral). The data on the diagonal of the matrix are the square root of the AVE, and the others are the corresponding correlation coefficients. The sample size was 451. PE: publicity and education; EV: environmental values; PS: price sensitivity; GCB: green consumption behavior.

**Table 4:** Results of the hypothesis test.

	Hypothesis			Estimate	SE	C.R.	*p*-Value
H1	EV	<---	PE	0.361	0.040	7.778	***
H2	GCB	<---	PE	0.179	0.060	2.996	**
H3	GCB	<---	EV	0.159	0.061	3.015	**

Note: ** *p* < 0.01, *** *p* < 0.001. SE: standard error; C.R.: critical ratio; H1: publicity and education (PE) positively affect environmental values (EV).H2: publicity and education positively (PE) affect individual green consumption behavior (GCB).H3: environmental values(EV) positively influence green consumption behavior (GCB).

**Table 5:** Results of the mediating effect test.

Path	Effects	Point Estimation	Bootstrap 95% CI		Support
			Lower	Upper	
PE ---> GCB	Total effect	0.372	0.253	0.494	Yes
	Indirect effect	0.130	0.040	0.231	Yes
	Direct effect	0.242	0.099	0.397	Yes

Note: PE: publicity and education; GCB: green consumption behavior.

**Table 6:** Test results of regulatory effect.

	EV		GCB	
	*β*	*t*	*β*	*t*
PE	0.368	10.272 ***	0.249	4.872 ***
EV			0.225	3.762 ***
PS	0.095	3.185 **		
PS ∗ PE	−0.202	−5.807 ***		
F-value	74.845 ***		38.649 ***	
R^2^	0.578		0.384	
R^2^ Adjusted	0.334		0.147	

Note: ** *p*< 0.01, *** *p* < 0.001. PE: publicity and education; EV: environmental values; PS: price sensitivity; GCB: green consumption behavior. R^2^: R square; PS ∗ PE: the multiplication of PS and PE.

**Table 7:** Test results from moderated effect.

Adjusting Variable Level	Estimate	LLCI	ULCI
−0.770	0.524	0.447	0.601
0.001	0.369	0.298	0.439
0.771	0.213	0.116	0.310

Note: LLCI: lower level confidence interval; ULCI: upper level confidence interval.

**Table 8:** Results from moderated mediating effect.

Adjusting Variable Level	Effect	LLCI	ULCI
−0.770	0.115	0.040	0.189
0.001	0.081	0.030	0.130
0.771	0.047	0.017	0.079

Note: LLCI: lower level confidence interval; ULCI: upper level confidence interval.

## Data Availability

The data that support the findings of this study are available onrequest from the corresponding author. The data are not publicly available due to privacy or ethicalrestrictions.

## Appendix A

*Publicity and education*

1. I often see news, publicity, and public service announcements about green environmental protection on TV and the Internet.
2. I have learned from the media and reports that the environmental problems caused by bad living habits are getting worse.
3. I know how to save and save energy from media propaganda and education.
4. Consumers pay more attention to ecological and environmental issues through education and publicity.

*Environmental values*

1. I think the current climate and environmental problems are quite serious.
2. The earth’s natural resources, such as energy, minerals, and forests, will run out if we do not conserve them.
3. The degradation of the climate and the environment is closely related to the daily lifestyle of human beings.
4. Garbage pollution has affected our living and living environment.
5. In order to survive, humans must live with nature in harmony.
6. Although humans have the power to transform nature, we still have to obey the laws of nature.

*Price sensitivity*

1. The main factor that determines whether I decide to buy green products is price.
2. Water and electricity prices continue to rise, so I pay more and more attention to saving water and electricity.
3. If the store has a special or reduced price on energy-saving products, I would buy them.

*Green consumption behavior*

1. To protect the environment, I try to buy energy-efficient appliances.
2. I don’t like over-packaged products, and I don’t usually buy them.
3. When buying a family car, I care more about energy saving and environmental protection.
4. In order to save energy, I usually accumulate enough dirty clothes before I use the washing machine.
5. When I get out, I prefer taking buses or walking to taking taxis.
6. I always bring my own shopping bags and try to avoid using disposable plastic bags.
7. When appliances (TV, air conditioner, etc.) are not used for a long time, I will cut off the power plug.
8. I usually collect old papers, unused books, etc. and then sell them together or give them away.
9. In my daily life, I will try to recycle products until they are completely discarded.
10. I will try my best to sort the garbage and put it into the dustbin.

## References

[1] Yang Y., Deng Y., Tuo Y., Li J., He T., Chen M. (2020). Study of the thermal regime of a reservoir on the Qinghai—Tibetan Plateau, China. PLoS ONE.

[2] Wang B., Jin H., Li Q., Chen D., Zhao L., Tang Y., Kato T., Gu S. (2017). Diurnal and seasonal variations in the net ecosystem CO_2_ exchange of a pasture in the three-river source region of the Qinghai—Tibetan Plateau. PLoS ONE.

[3] Zhao Y., Zhang L. (1999). Study on method of quantitative assessment of fragile environment. Chin. Geogr. Sci..

[4] Fan J., Xu Y., Wang C., Niu Y., Chen D., Sun W. (2015). The effects of human activities on the ecological environment of Tibet over the past half century. Chin. Sci. Bull..

[5] Hoekstra A.Y., Wiedmann T.O. (2014). Humanity’s unsustainable environmental footprint. Science.

[6] Chen J., Li T., Zhang Y. (2017). Growing season carries stronger contributions to albedo dynamics on the Tibetan plateau. PLoS ONE.

[7] Schipper L., Bartlett S., Hawk D., Vine E. (1989). Linking Life-Styles and Energy Use: A Matter of Time?. Annu. Rev. Energy.

[8] Liobikienė G., Bernatonienė J. (2017). Why determinants of green purchase cannot be treated equally? The case of green cosmetics: Literature review. J. Clean. Prod..

[9] Prothero A., Dobscha S., Freund J., Kilbourne W.E., Luchs M.G., Ozanne L.K., Thøgersen J. (2011). Sustainable consumption: Opportunities for consumer research and public policy. J. Public Policy Mark..

[10] Guo Y., Zhang P., Liao J., Wu F. (2020). Social Exclusion and Green Consumption: A Costly Signaling Approach. Front. Psychol..

[11] Ge W., Sheng G., Zhang H. (2020). How to Solve the Social Norm Conflict Dilemma of Green Consumption: The Moderating Effect of Self-Affirmation. Front. Psychol..

[12] Amatulli C., De Angelis M., Peluso A.M., Soscia I., Guido G. (2019). The effect of negative message framing on green consumption: An investigation of the role of shame. J. Bus. Ethics.

[13] Raukoff, Wu J. (2013). Influence Mechanism of Green Consumption Behavior Based on Ajzen Planned Behavior Theory. J. Financ. Econ..

[14] Chen K., Guo F., Zhao Z.B. (2013). Mechanism Analysis of Psychological Factors of Green Consumption Behavior: From the Research Perspective of Psychological Process of Green Consumption Behavior. Enterp. Econ..

[15] Li M. (2020). Review of Consumers’ Green Consumption Behavior. Am. J. Ind. Bus. Manag..

[16] ElHaffar G., Durif F., Dubé L. (2020). Towards closing the attitude-intention-behavior gap in green consumption: A narrative review of the literature and an overview of future research directions. J. Clean. Prod..

[17] Follows S.B., Jobber D. (2000). Environmentally responsible purchase behaviour: A test of a consumer model. Eur. J. Mark..

[18] Van Vugt M. (2009). Averting the tragedy of the commons: Using social psychological science to protect the environment. Curr. Dir. Psychol. Sci..

[19] Matsukawa I. (2000). Household response to incentive payments for load shifting: A Japanese time-of-day electricity pricing experiment. Energy J..

[20] Holland R.W., Verplanken B., Van Knippenberg A. (2002). On the nature of attitude–behavior relations: The strong guide, the weak follow. Eur. J. Soc. Psychol..

[21] Johnstone M.-L., Hooper S. (2016). Social influence and green consumption behaviour: A need for greater government involvement. J. Mark. Manag..

[22] Mishal A., Dubey R., Gupta O.K., Luo Z. (2017). Dynamics of environmental consciousness and green purchase behaviour: An empirical study. Int. J. Clim. Chang. Strateg. Manag..

[23] Barber N.A., Bishop M., Gruen T. (2014). Who pays more (or less) for pro-environmental consumer goods? Using the auction method to assess actual willingness-to-pay. J. Environ. Psychol..

[24] Liu Y., Lu C. (2021). Quantifying Grass Coverage Trends to Identify the Hot Plots of Grassland Degradation in the Tibetan Plateau during 2000–2019. Int. J. Environ. Res. Public Health.

[25] Li S., Wu J., Gong J., Li S. (2018). Human footprint in Tibet: Assessing the spatial layout and effectiveness of nature reserves. Sci. Total Environ..

[26] Li S., Zhang Y., Wang Z., Li L. (2018). Mapping human influence intensity in the Tibetan Plateau for conservation of ecological service functions. Ecosyst. Serv..

[27] Chong A.C., Chu A.M., So M.K., Chung R.S. (2019). Asking sensitive questions using the randomized response approach in public health research: An empirical study on the factors of illegal waste disposal. Int. J. Environ. Res. Public Health.

[28] Xue-Yong Z., Sen L., Chun-Lai Z., Guang-Rong D., Yu-Xiang D. (2002). Desertification and control plan in the Tibet Autonomous Region of China. J. Arid Environ..

[29] Gyberg P., Palm J. (2009). Influencing households’ energy behaviour—How is this done and on what premises?. Energy Policy.

[30] Kirakozian A. (2016). The determinants of household recycling: Social influence, public policies and environmental preferences. Appl. Econ..

[31] Corraliza J.A., Berenguer J. (2000). Environmental values, beliefs, and actions: A situational approach. Environ. Behav..

[32] Gilg A., Barr S., Ford N. (2005). Green consumption or sustainable lifestyles? Identifying the sustainable consumer. Futures.

[33] Shun-xin X.K.-x.Y. (2007). Introduction to Tibetan Ecological Ethics. J. Qinghai Natl. Inst..

[34] Li D. (2019). A Scientific Interpretation of the Complexity of Tibetan Ecological Ethics. Proceedings of the 3rd International Conference on Art Studies: Science, Experience, Education (ICASSEE 2019), Moscow, Russia, 4–5 October 2019.

[35] Zhou X., Liu Y., Ho B. (2015). The cultural transmission of cooperative norms. Front. Psychol..

[36] Hines J.M., Hungerford H.R., Tomera A.N. (1987). Analysis and synthesis of research on responsible environmental behavior: A meta-analysis. J. Environ. Educ..

[37] Stern P. (2000). Toward a coherent theory of environmentally significant behavior. J. Soc. Issues.

[38] Kichikawa Y., Iyetomi H., Aoyama H., Fujiwara Y., Yoshikawa H. (2020). Interindustry linkages of prices—Analysis of Japan’s deflation. PLoS ONE.

[39] Adena M., Huck S., Rasul I. (2017). Testing consumer theory: Evidence from a natural field experiment. J. Econ. Sci. Assoc..

[40] Sinha I., Batra R. (1999). The effect of consumer price consciousness on private label purchase. Int. J. Res. Mark..

[41] Lichtenstein D.R., Ridgway N.M., Netemeyer R.G. (1993). Price perceptions and consumer shopping behavior: A field study. J. Mark. Res..

[42] Cicia G., Del Giudice T., Ramunno I. (2009). Environmental and health components in consumer perception of organic products: Estimation of willingness to pay. J. Food Prod. Mark..

[43] Gleim M.R., Smith J.S., Andrews D., Cronin J.J. (2013). Against the green: A multi-method examination of the barriers to green consumption. J. Retail..

[44] Sardianou E. (2007). Estimating energy conservation patterns of Greek households. Energy Policy.

[45] Lee K. (2010). The green purchase behavior of Hong Kong young consumers: The role of peer influence, local environmental involvement, and concrete environmental knowledge. J. Int. Consum. Mark..

[46] Dunlap R.E. (2008). The new environmental paradigm scale: From marginality to worldwide use. J. Environ. Educ..

[47] Van Doorn J., Verhoef P.C. (2015). Drivers of and barriers to organic purchase behavior. J. Retail..

[48] Lee Y.-K., Choi J.-G., Kim M.-S., Ahn Y.-G., Katz-Gerro T. (2012). Explaining pro-environmental behaviors with environmentally relevant variables: A survey in Korea. Afr. J. Bus. Manag..

[49] Fan Y., Chen J., Shirkey G., John R., Wu S., Park H., Shao C. (2016). Applications of structural equation modeling (SEM) in ecological studies: An updated review. Ecol. Process..

[50] Hovland C.I. (1954). Communication and persuasion. J. Consult. Psychol..

[51] Han J., Seo Y., Ko E. (2017). Staging luxury experiences for understanding sustainable fashion consumption: A balance theory application. J. Bus. Res..

[52] Røpke I. (1999). The dynamics of willingness to consume. Ecol. Econ..

[53] Boiral O., Talbot D., Paillé P. (2015). Leading by example: A model of organizational citizenship behavior for the environment. Bus. Strategy Environ..

[54] Mostafa M.M. (2016). Post-materialism, religiosity, political orientation, locus of control and concern for global warming: A multilevel analysis across 40 nations. Soc. Indic. Res..

[55] Kacen J.J., Lee J.A. (2002). The influence of culture on consumer impulsive buying behavior. J. Consum. Psychol..

[56] Pope C.A., Dockery D.W. (2006). Health effects of fine particulate air pollution: Lines that connect. J. Air Waste Manag. Assoc..

[57] Khezri B., Chan Y.Y., Tiong L., Webster R.D. (2015). Annual air pollution caused by the Hungry Ghost Festival. Environ. Sci. Process. Impacts.

[58] Van den Bergh J.C., Ferrer-i-Carbonell A. (1999). Economic Theories of Sustainable Consumption.

[59] Weisstein F.L., Asgari M., Siew S.-W. (2014). Price presentation effects on green purchase intentions. J. Prod. Brand Manag..

[60] Litvine D., Wüstenhagen R. (2011). Helping “light green” consumers walk the talk: Results of a behavioural intervention survey in the Swiss electricity market. Ecol. Econ..

[61] Wijekoon R., Sabri M.F. (2021). Determinants That Influence Green Product Purchase Intention and Behavior: A Literature Review and Guiding Framework. Sustainability.

[62] Barbarossa C., Pastore A. (2015). Why environmentally conscious consumers do not purchase green products. Qual. Mark. Res..

